# HE4 promotes collateral resistance to cisplatin and paclitaxel in ovarian cancer cells

**DOI:** 10.1186/s13048-016-0240-0

**Published:** 2016-05-17

**Authors:** J. R. Ribeiro, C. Schorl, N. Yano, N. Romano, K. K. Kim, R. K. Singh, R. G. Moore

**Affiliations:** Women and Infants Hospital, Department of Obstetrics and Gynecology, Program in Women’s Oncology, Molecular Therapeutics Laboratory, 200 Chestnut Street, Providence, RI 02903 USA; Center for Genomics and Proteomics, Genomics Core Facility, Brown University, 70 Ship Street, Providence, RI 02903 USA; Wilmot Cancer Institute, Division of Gynecologic Oncology, Department of Obstetrics and Gynecology, University of Rochester Medical Center, Rochester, NY USA

**Keywords:** HE4, Epithelial ovarian cancer, Chemoresistance, Paclitaxel resistance, Platinum resistance, MAPK signaling, Tubulin, EGR1, SKOV3 cells

## Abstract

**Background:**

Chemotherapy resistance presents a difficult challenge in treating epithelial ovarian cancer patients, particularly when tumors exhibit resistance to multiple chemotherapeutic agents. A few studies have shown that elevated serum levels of the ovarian cancer biomarker HE4 correlate with tumor chemoresistance, response to treatment, and survival. Here, we sought to confirm our previous results that HE4 contributes to collateral resistance to cisplatin and paclitaxel in vitro and uncover factors that may contribute to HE4-mediated chemoresistance.

**Methods:**

MTS assays and western blots for cleaved PARP were used to assess resistance of HE4-overexpressing SKOV3 and OVCAR8 clones to cisplatin and paclitaxel. CRISPR/Cas technology was used to knockdown HE4 in HE4-overexpressing SKOV3 cells. A microarray was conducted to determine differential gene expression between SKOV3 null vector-transfected and HE4-overexpressing clones upon cisplatin exposure, and results were validated by quantitative RT-PCR. Regulation of mitogen activated protein kinases (MAPKs) and tubulins were assessed by western blot.

**Results:**

HE4-overexpressing SKOV3 and OVCAR8 clones displayed increased resistance to cisplatin and paclitaxel. Knockdown of HE4 in HE4-overexpressing SKOV3 cells partially reversed chemoresistance. Microarray analysis revealed that HE4 overexpression resulted in suppression of cisplatin-mediated upregulation of *EGR1*, a MAPK-regulated gene involved in promoting apoptosis. Upregulation of p38, a MAPK activated in response to cisplatin, was suppressed in HE4-overexpressing clones. No differences in extracellular signal-regulated kinase (ERK) activation were noted in HE4-overexpressing clones treated with 25 μM cisplatin, but ERK activation was partially suppressed in HE4-overexpressing clones treated with 80 μM cisplatin. Furthermore, treatment of cells with recombinant HE4 dramatically affected ERK activation in SKOV3 and OVCAR8 wild type cells. Recombinant HE4 also upregulated α-tubulin and β-tubulin levels in SKOV3 and OVCAR8 cells, and microtubule associated protein tau (*MAPT*) gene expression was increased in SKOV3 HE4-overexpressing clones.

**Conclusions:**

Overexpression of HE4 promotes collateral resistance to cisplatin and paclitaxel, and downregulation of HE4 partially reverses this chemoresistance. Multiple factors could be involved in HE4-mediated chemoresistance, including deregulation of MAPK signaling, as well as alterations in tubulin levels or stability.

**Electronic supplementary material:**

The online version of this article (doi:10.1186/s13048-016-0240-0) contains supplementary material, which is available to authorized users.

## Background

Chemotherapy resistance is a challenging problem in the treatment of epithelial ovarian cancer (EOC) patients. While a majority (80 %) of women initially responds to first-line platinum/taxane chemotherapy, recurrent disease presents in 60–85 % of patients, and is fundamentally incurable [1, 2]. However, patients with recurrent disease that is platinum-sensitive have a better response rate and improved progression free survival (PFS) and overall survival (OS) when treated with combination therapy [2]. In patients with platinum-resistant disease, single agent paclitaxel was shown to produce an objective response rate of only 22–30 % [2]. In the case of patients with disease that is resistant to both platinum and paclitaxel, other options are available, such as pegylated liposomal doxorubicin (PLD), topotecan, and gemcitabine [2]; however, it is not always clear which patients may benefit most from each therapy. Ultimately, although multidrug regimens are associated with higher toxicity, they are more effective than single agent therapies. Gaining a better understanding of factors contributing to resistance to platinum and taxane based therapies will be valuable in guiding treatment decisions for ovarian cancer patients.

Human epididymis protein-4 (HE4/WFDC2) is a small secretory protein that belongs to the family of whey acidic protein (WAP) domain-containing anti-proteases [3], and has been shown to possess cross-class anti-protease activity itself [4]. It is overexpressed in ovarian cancer tissues compared to normal ovaries, and has been identified as a novel biomarker for EOC [5, 6]. Serum levels predict ovarian cancer with equivalent sensitivity to CA125, but with the advantage that HE4 is less frequently elevated in patients with benign gynecological conditions [5]. A multicenter study led by our institution established a new algorithm for the diagnosis of women with an ovarian mass [6]. The FDA-approved Risk of Ovarian Malignancy Algorithm (ROMA) uses HE4 along with CA125 and menopausal status to predict a woman’s risk of ovarian cancer and monitor disease with improved sensitivity and specificity over the Risk of Malignancy Index (RMI) that used CA125, pelvic sonography, and menopausal status [6].

Recently, HE4 has been associated with the development of chemoresistance clinically. We have previously determined that HE4 and ROMA scores are more sensitive predictors of platinum response than CA125 alone [7]. Angioli et al. reported that serum HE4 levels predict platinum-resistant versus sensitive disease at the third chemotherapy cycle with 100 % sensitivity and 85 % specificity [8]. Similarly, another study found that serum HE4 concentration is higher in patients resistant to first-line chemotherapy [9]. Among high-grade serous ovarian cancer (HGSC) patients, those whose HE4 levels displayed greater reduction during neoadjuvant chemotherapy had improved OS [10]. Collectively, these studies point to HE4 as a predictor of chemotherapy response/resistance, but do not address the question of whether HE4 has a causative role in the development of resistance.

We have also shown that SKOV3 ovarian cancer cells overexpressing HE4 are more resistant to cisplatin and paclitaxel, while HE4-overexpressing OVCAR8 ovarian cancer cells exhibit greater cisplatin resistance than their null vector-transfected counterparts [7]. In mice, SKOV3 xenografts overexpressing HE4 also grew larger than control SKOV3 xenografts [7]. In support of these data, Wang et al. found that recombinant HE4-treated SKOV3 cells display reduced carboplatin-induced apoptosis, a decreased ratio of *BAX*/*BCL2*, and an overall downregulation of genes involved in DNA damage response and apoptosis [11]. However, much remains to be elucidated regarding how HE4 promotes resistance to platinum or taxane therapies. Therefore, we sought first to confirm our preliminary studies suggesting a causative role for HE4 in cisplatin and paclitaxel resistance. Our second goal was to examine the gene expression profile of SKOV3 cells overexpressing HE4, as well as determine differences in regulation of gene expression in response to cisplatin treatment. Herein, we also begin to explore a few of the multifold processes that may contribute to HE4-mediated chemoresistance.

## Methods

### Cell culture

SKOV3 and OVCAR8 ovarian cancer cells were obtained from American Type Culture Collection (Manassas, VA). Cells were cultured in Dulbecco Modified Eagle Medium (DMEM, Gibco, 11965-065) with 10 % fetal bovine serum (FBS) and 1 % penicillin/streptomycin and kept in a 37 °C humidified incubator with 5 % CO^2^.

### Stable cell lines

All null vector (NV) and HE4-overexpressing stable cell lines were previously established [7]. To generate HE4-CRISPR Double Nickase stable cell lines, SKOV3-C1 cells were transfected in 6-well plates with 1 μg HE4 Double Nickase Plasmid (Santa Cruz, sc-402876-NIC) or Control Double Nickase Plasmid (Santa Cruz, sc-437281), using 5 μl Lipofectamine 2000 (Invitrogen). After 48 h, media was changed to 1 μg/ml puromycin containing media for five days, then split into larger plates and selected for an additional five days. RNA and tissue culture supernatant was collected to confirm downregulation of HE4 levels by quantitative RT-PCR (qRT-PCR) and ELISA. Cells were maintained in DMEM supplemented with 10 % FBS, 1 % penicillin/streptomycin, and 1 μg/ml puromycin.

### Cell treatments

Cells were treated with the described doses of cis-diamminedichloroplatinum(II) (cisplatin, Sigma Aldrich, 1134357) or paclitaxel (Sigma Aldrich, T7402) dissolved in dimethyl sulfoxide (DMSO, Sigma Aldrich, D8418), or DMSO alone as a control. Cells were collected directly into either Trizol (Ambion, 15596018) or Cell Lysis Buffer (Cell Signaling, 9803) at the indicated time points for analysis. Cells were treated with recombinant human HE4 (rHE4, MyBioSource, MBS355616) added directly to the media to a final concentration of 20 nM. Cells were treated with recombinant human epidermal growth factor (rEGF, Calbiochem, 324831) at a concentration of 10 ng/ml.

### Cell viability assays

All cells were seeded at 2 000 cells/well in 96-well plates. Cells were treated with increasing doses of cisplatin and paclitaxel as described. After 48 h, cell viability assays were performed by adding 10 μl/well of CellTiter 96® Aqueous One Solution Cell Proliferation MTS Assay (Promega, G3580), incubating at 37 °C/5 % CO^2^ for 2 h, and reading absorbance at 492 nm. Results are displayed as percent survival of vehicle treated cells.

### Microarray

SKOV3-NV and SKOV3-C1 cells were treated in triplicate at 80 % confluency with 25 μM cisplatin or DMSO vehicle. Total RNA was collected 24 h later using an RNeasy Mini Kit (Qiagen, 74104) and checked for purity by NanoDrop 2000 (Thermo Scientific). The RNA samples were randomly assigned numbers and submitted to the Brown Genomics Core Facility for Bioanalyzer (Agilent 2100) RNA quality analysis. Affymetrix HTA 2.0 Arrays were performed according to the manufacturer’s instructions at the Core Facility using 150 ng total input RNA.

### DAVID analysis

Database for Annotation, Visualization, and Integrated Discovery (DAVID) v6.7 [12, 13] was used to identify the top ten enriched annotation terms among 180 genes differentially expressed (1.5-fold in either direction, *p* < .05) between SKOV3-NV and SKOV3-C1. Default DAVID parameters were employed as follows:

Kappa Similarity: Similarity Term Overlap – 3; Similarity Threshold – 0.5

Classification: Initial Group Membership – 3; Final Group Membership – 3; Multiple Linkage Threshold – 0.5

Enrichment Threshold: EASE – 1.0

Stringency: Medium

### Quantitative PCR

RNA was collected using an RNeasy Mini Kit (Qiagen, 74104) or Trizol extraction/LiCl precipication. Total RNA (500 ng) was reverse transcribed into cDNA using the iScript cDNA Synthesis Kit (Bio-Rad, 1708890) according the manufacturer’s protocol. To validate differentially expressed genes between SKOV3-NV and SKOV3-C1 cells identified by microarray, the same RNA samples used for the microarray were employed. To validate the differential cisplatin-induced upregulation of *EGR1* between SKOV3-NV and SKOV3-C1/C7, microarray RNA samples were used, as well as RNA isolated from SKOV3-C7 cells that were treated in the same manner as the cells used in the microarray. Quantitative PCR was performed in triplicate by loading 1 μl cDNA reaction, 2 μl each of 5 μM custom forward and reverse primers (Invitrogen) or 1 μM forward and reverse validated primers (realtimeprimers.com), 10 μl SYBR Green (Applied Biosciences [ABI], 4367659) and 5 μl RNAse-free water to each well. Samples were run on an ABI 7500 Fast Real-Time PCR System, and data was analyzed using the ΔΔCt method. Relative expression levels were normalized to 18 s rRNA to correct for equivalent total RNA levels. Validated *MAPT, CYP1B1,* and *EGR1* primers were purchased from realtimeprimers.com. Custom primer sequences (Invitrogen) are as follows:

*AKT3* F – AAG GGA AGA ATG GAC AGA

*AKT3* R – ATG GGT TGT AGA GGC ATC

*NMUR2* F – CCG TTC CAC ATT GAC CGA CT

*NMUR2* R – CAC CAC ATG GAC GAG GTT GA

*SEPT3* F – TTG CCC TGC TTC GAG ACT TT

*SEPT3* R – CTT TCC TCT GTG TCC ACG CT

18 s rRNA F – CCG CGG TTC TAT TTT GTT GG

18 s rRNA R – GGC GCT CCC TCT TAA TCA TG

### Western blot

Protein was extracted from cell pellets in Cell Lysis Buffer (Cell Signaling, 9803) with 1 mM PMSF, according to the manufacturer’s protocol. Protein concentrations were determined by DC Protein Assay (Bio-Rad Laboratories, 5000116). Western blot analysis was performed by loading equal amounts of protein boiled with Novex Sample Reducing Agent (Life Technologies, NP009) and NuPAGE LDS sample buffer (ThermoFisher Scientific, NP0007) into a 4–12 % gradient NuPAGE Novex Bis-Tris gel [Life Technologies, NP0321BOX (mini), WG1402BX10 (midi)]. Protein was transferred by semi-dry transfer to methanol-activated 0.2 μm PVDF membranes (Bio-Rad, 162-0177) at 0.12-0.2 A for 1 h 15 m. Membranes were blocked in 5 % milk in phosphate-buffered saline with 0.05 % Tween 20 (PBS-T) for 30 m at room temperature, incubated in primary antibody in 5 % milk in PBS-T overnight at 4 °C, and then in secondary antibody in 5 % milk in PBS-T for 1 h at room temperature, with PBS-T washes in between. Amersham ECL Prime Western Blot Detection System (GE Healthcare, RPN2232) was used for detection of HRP-tagged secondary antibodies. Blots were developed using x-ray film in a Kodac film developer or imaged directly in a Biorad Chemidoc MP Imaging System. GAPDH was used as a loading control. Antibodies and dilutions used are as follows:

PARP (Cell Signaling, 9532, 1:1000)

phospho-p44/42 MAPK (ERK1/2) (Cell Signaling, 4370, 1:2000)

p44/42 (ERK1/2) (Cell Signaling, 9102, 1:2000)

EGR1 (Santa Cruz, sc-110, 1:200)

p38 (Cell Signaling, 9212, 1:1000)

phospho-p38 (Cell Signaling, 9215, 1:1000)

GAPDH (Cell Signaling, 2118, 1:2000)

β-tubulin (Cell Signaling, 2146, 1:2000)

α-tubulin (Cell Signaling, 2144, 1:1000)

### Densitometry

Image J was used to perform densitometry analysis of western blots. Images of blots were analyzed in 8-bit TIFF format, using the “analyze gel” function. Where no band was detected, a value of “1” was assigned. Relative band densities were normalized to a loading control, or the appropriate total protein for phospho-proteins, and then the lowest value was set to 1.

### Statistics

In all instances where statistics are shown, they represent n ≥ 3 independent experiments, and *p*-values were determined by unpaired 2-tailed Student *t*-test. For the microarray, Affymetrix Transcriptome Analysis Console (TAC) software was used to generate fold-changes and statistical significance, and ANOVA *p*-values generated by TAC were used for *p*-value cutoffs.

## Results

### HE4-overexpressing cells are more resistant to cisplatin and paclitaxel treatment

**Fig. 1:**
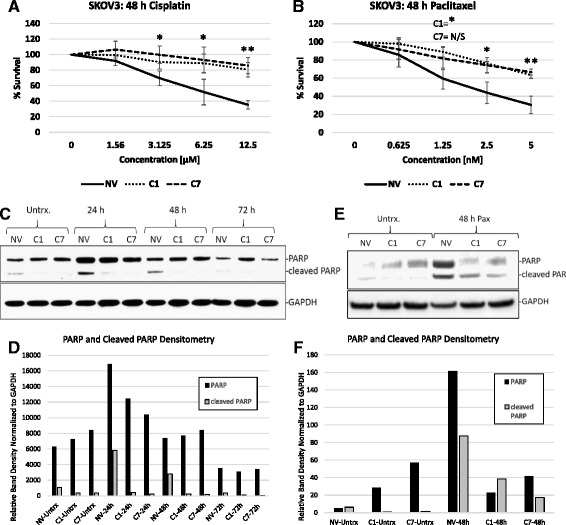
HE4-overexpressing cells are more resistant to cisplatin and paclitaxel treatment. SKOV3-NV, SKOV3-C1, and SKOV3-C7 cells were treated with 0-12.5 μM cisplatin (**a**) or 0-5 nM paclitaxel (**b**) for 48 h, at which time the cells were subjected to MTS assay to measure viability. Results are displayed as percent survival of vehicle treated cells. Error bars represent the standard deviation of biological replicates. *p < .05, **p < .005, N/S = not significant (**c**) SKOV3-NV, SKOV3-C1, and SKOV3-C7 cells were treated with vehicle (DMSO) or 5 μM cisplatin, and protein was collected at 24, 48, and 72 h after treatment. Western blot was performed to detect levels of PARP and cleaved PARP. **d** Densitometry analysis of PARP and cleaved PARP normalized to GAPDH, from western blot in (**c**). **e** SKOV3-NV, SKOV3-C1, and SKOV3-C7 cells were treated with 1 nM paclitaxel and protein was isolated from all cells (floating and adherent) 48 h after treatment. Western blot was performed to detect levels of PARP and cleaved PARP. GAPDH was used as a loading control. **f** Densitometry analysis of PARP and cleaved PARP normalized to GAPDH, from western blot in (**e**)

Since HE4 serum levels correlate with cisplatin resistance in ovarian cancer patients [7–10], and our previous data suggested that HE4 promotes chemotherapy resistance in vitro and in vivo [7], we set out to further define the chemotherapy response of ovarian cancer cells that overexpress HE4. We employed SKOV3 ovarian cancer cells stably expressing a null vector plasmid (SKOV3-NV) and two different clonally-selected cell lines stably expressing a pCMV6-HE4 plasmid (SKOV3-C1 and SKOV3-C7) [7]. SKOV3 cells are ideal to examine the effects of HE4 overexpression since they secrete very low levels of HE4 (<15 pM), as detected by ELISA [7]. We treated the cells with 0, 1.56, 3.125, 6.25, and 12.5 μM cisplatin or 0, 0.625, 1.25, 2.5, and 5 nM paclitaxel for 48 h, then performed MTS assays to measure cell viability. SKOV3-C1 and SKOV3-C7 cells were significantly more resistant than SKOV3-NV cells to cisplatin (Fig. 1a) at 3.125 μM (*p* = .029 and .024, respectively), 6.25 μM (*p* = .034 and 0.37, respectively), and 12.5 μM (*p* = .0020 and .0018, respectively). SKOV3-C1 and SKOV3-C7 cells were also more resistant than SKOV3-NV cells to paclitaxel (Fig. 1b) at 1.25 nM (*p* = .017 and .083, respectively), 2.5 nM (*p* = .0094 and .022, respectively) and 5 nM (*p* = .0050 and .0035, respectively).

In order to confirm that this resistance was due to inhibition in apoptosis, we analyzed the cleavage of poly ADP ribose polymerase (PARP) in SKOV3-NV, SKOV3-C1, and SKOV3-C7 cells treated with 5 μM cisplatin for 24, 48, and 72 h. Even in untreated cells, baseline levels of cleaved PARP were lower in HE4-overexpressing clones than in SKOV3-NV cells. In SKOV3-NV cells, PARP cleavage was clearly increased by 5 μM cisplatin at 24 h, and continued to remain elevated at 48 h. By 72 h, levels of cleaved PARP had diminished in SKOV3-NV cells, presumably because most of the cells had already undergone apoptosis. In HE4-overexpressing clones, uncleaved PARP did accumulate to a similar degree as in SKOV3-NV cells—suggesting that the cisplatin was able to enter the cells and cause DNA damage, thus recruiting PARP to sites of single-strand breaks. However, cleavage of PARP did not occur in HE4-overexpressing cells, indicating that the process of apoptosis was not completed (Fig. 1c-d). We also observed an accumulation in both PARP and cleaved PARP in SKOV3-NV cells treated with 1 nM paclitaxel for 48 h, but did not see this response as strongly in SKOV3-C1 or SKOV3-C7 cells (Fig. 1e-f). The fact that PARP did not accumulate robustly in HE4-overexpressing clones treated with paclitaxel—unlike what we saw with cisplatin treatment—is likely due to the vastly different mechanisms of action by which these two drugs result in DNA damage.

Preliminary dose response experiments revealed that OVCAR8 cells required high doses of cisplatin to achieve cell death. Thus, we treated OVCAR8-NV and HE4-overexpressing clones (OVCAR8-C5) with 0, 62.5, 125, 250, and 500 μM cisplatin for 24 h, and found a slight increase in resistance in the OVCAR8-C5 cells (Additional file 1A), which was also reflected by lower levels of cleaved PARP in OVCAR8-C5 cells than OVCAR8-NV cells treated with 100 μM cisplatin for 24 h (Additional file 1B-C). However, although OVCAR8 cells also secrete undetectable levels of HE4 as determined by ELISA (data not shown), they appear to already be highly resistant to cisplatin and are not an ideal model to examine the effects of HE4 on chemotherapy resistance. Likewise, OVCAR8-NV cells were also more resistant to paclitaxel than SKOV3-NV cells, and only exhibited a modest increase in resistance with HE4 overexpression (Additional file 1D). For this reason, we focused primarily on SKOV3 cells for the remainder of our studies.

### CRISPR/Cas-mediated HE4 downregulation reduces chemoresistance in SKOV3-C1 cells

**Fig. 2:**
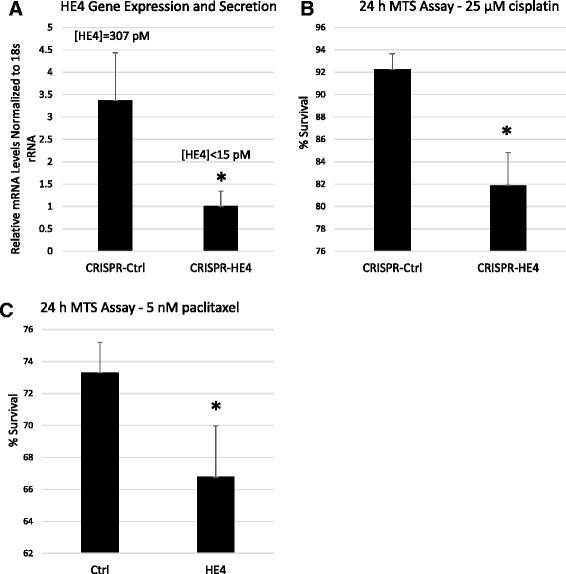
CRISPR/Cas-mediated HE4 downregulation reduces chemoresistance in SKOV3-C1 cells. **a** SKOV3-C1 cells were transfected with a human CRISPR-HE4 Double Nickase plasmid and CRISPR-Control Double Nickase plasmid for 48 h. Transfected cells were selected with puromycin and efficient downregulation of HE4 was measured by qRT-PCR and ELISA. [HE4] = concentration of HE4 in supernatant as measured by ELISA. **b**-**c** CRISPR-Control and CRISPR-HE4 cells were treated with DMSO vehicle or 25 μM cisplatin or 5 nM paclitaxel for 24 h, and MTS assay was performed to measure cell viability. Results are displayed as percent survival of vehicle treated cells. Error bars represent the standard deviation of three or more biological replicates, *p < .05

In order to confirm that overexpression of HE4 was responsible for the increase in cisplatin resistance observed, we knocked down HE4 in SKOV3-C1 cells by stable transfection of a human HE4 CRISPR/Cas Double Nickase plasmid. RNA from HE4-targeted cells (CRISPR-HE4) and control plasmid transfected cells (CRISPR-Ctrl) was collected to confirm effective targeting of the HE4 gene. Cell culture supernatant was also collected and subjected to ELISA to determine levels of secreted HE4 in the media. CRISPR-HE4 transfected cells displayed 70.3 % knockdown (*p* = .02) in HE4 mRNA levels compared to control cells. Furthermore, CRISPR-HE4 media had undetectable levels (<15 pM) of HE4, which is in agreement with what we previously observed in SKOV3 wild type and SKOV3-NV cells. HE4 concentration in CRISPR-Ctrl media measured 307 pM, which was comparable to our previously reported levels for SKOV3-C1 and SKOV3-C7 cells (250 pM and 362 pM, respectively) [7] (Fig. 2a). Next, we measured survival of CRISPR-Ctrl and CRISPR-HE4 cells in response to 25 μM cisplatin treatment (24 h), and found that CRISPR-Ctrl cells exhibited 92.2 % cell survival compared to vehicle-treated cells, while CRISPR-HE4 cells displayed 81.9 % survival compared to vehicle-treated cells (*p* = .005; Fig. 2b). Likewise, CRISPR-Ctrl cells exhibited 73.9 % survival in response to paclitaxel versus 65.9 % survival of CRISPR-HE4 cells (*p* = .005) (Fig. 2c).

### Microarray analysis reveals genes differentially regulated by HE4 in ovarian cancer cells

**Fig. 3:**
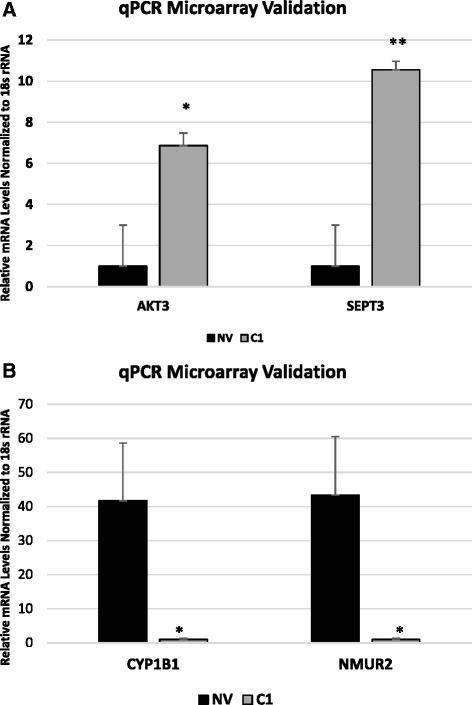
Microarray qPCR validation. *AKT3* and *SEPT3* (**a**), and *CYP1B1* and *NMUR2* (**b**) were selected to validate microarray results by quantitative RT-PCR. Error bars represent the standard deviation of three biological replicates, *p < .05, **p < .005

In order to gain a better understanding of the differential regulation of transcription between SKOV3-NV and SKOV3-C1 cells in the presence and absence of cisplatin, we performed a microarray analysis of total RNA collected from SKOV3-NV and SKOV3-C1 cells treated with either vehicle [dimethylsulfoxide (DMSO)] or 25 μM cisplatin for 24 h (*n* = 3/group). All transcripts differentially regulated between groups 1.5-fold in either direction (ANOVA *p*-value < .05) are available in Additional file 2. The top fifteen annotated, protein-coding genes that were differentially regulated in vehicle-treated SKOV3-C1 cells compared to SKOV3-NV (*p* < .05) are shown in Table 1. The Database for Annotation, Visualization and Integrated Discovery (DAVID) [12, 13] was used to perform gene ontology analysis of all genes differentially expressed 1.5-fold in either direction (*p* < .05) in SKOV3-C1 cells versus SKOV3-NV. This analysis revealed enrichment for the terms “secreted glycoproteins”, “transmembrane domains”, “pregnancy and immunoglobulin-like domains”, “cell adhesion”, “plasma membrane”, “fibronectin”, “insulin-like growth factor binding”, “membrane fraction”, “extracellular matrix”, and “EGF-like domains” (Additional file 3). We validated four of the genes from the list of most differentially expressed genes by quantitative reverse-transcription PCR (qRT-PCR), as follows: V-Akt Murine Thymoma Viral Oncogene Homolog 3 (*AKT3)*; septin 3 (*SEPT3*); cytochrome p450, family 1, subfamily B, polypeptide 1 (*CYP1B1*); and neuromedin 2 (*NMUR2*) (Fig. 3a-b).

**Table 1:** Genes differentially expressed between SKOV3-NV and SKOV3-C1 (vehicle treated). SKOV3-NV and SKOV3-C1 cells were treated with vehicle (DMSO) for 24 h (*n* = 3/group). RNA was isolated and Affymetrix HTA 2.0 arrays were performed to determine differences in gene expression between SKOV3-NV and SKOV3-C1 cells. The top fifteen annotated, protein-coding genes (*p* < .05) in either direction are listed below

Genes differentially expressed between SKOV3-NV and SKOV3-C1 (vehicle treated)			
Gene symbol	Description	Fold change (NV/C1)	ANOVA *p*-value
CYP1B1	Cytochrome P450, family 1, subfamily B, polypeptide 1	14.95	.000009
LAPTM5	Lysosomal protein transmembrane 5	12.8	2.58E-08
ANPEP	Alanyl (membrane) aminopeptidase	12.09	1.27E-08
NMUR2	Neuromedin U receptor 2	11.96	.000004
EPHA7	EPH receptor A7	6.69	.000015
PKP2	Plakophilin 2	5.56	.000008
SLC43A3	Solute carrier family 43, member 3	5	.000012
ACSM3	Acyl-CoA synthetase medium-chain family member 3	4.62	.00002
POF1B	Premature ovarian failure, 1B	3.98	.000026
CTH	Cystathionase (cystathionine gamma-lyase)	3.76	.000036
ALPK2	Alpha-kinase 2	3.55	.000021
KCTD4	Potassium channel tetramerisation domain containing 4	3.47	.000099
EREG	Epiregulin	3.42	.000006
DPP4	Dipeptidyl-peptidase 4	3.31	.000635
IL1A	(interleukin 1, alpha)	3.19	0.000206
AKT3	v-akt murine thymoma viral oncogene homolog 3 (protein kinase B, gamma)	-4.03	.000163
RRAGD	Ras-related GTP binding D	-4.06	.000313
PSG3	Pregnancy specific beta-1-glycoprotein 3	-4.09	2.66E-07
POPDC3	Popeye domain containing 3	-4.18	.000124
SH3BGRL2	SH3 domain binding glutamic acid-rich protein like 2	-4.29	.000331
OLFM2	Olfactomedin 2	-4.33	.002074
DPYSL5	Dihydropyrimidinase-like 5	-4.48	.000457
SEPT3	Septin 3	-4.57	.000203
S1PR1	Sphingosine-1-phosphate receptor 1	-4.6	.000134
DSC3	Desmocollin 3	-5.13	.000179
GPC4	Glypican 4	-5.22	.000063
ADAM23	ADAM metallopeptidase domain 23	-5.53	.000245
PDE3B	Phosphodiesterase 3B, cGMP-inhibited	-6.63	.000081
PSG1	Pregnancy specific beta-1-glycoprotein 1	-6.73	.000064
CYP24A1	Cytochrome P450, family 24, subfamily A, polypeptide 1	-7.34	.000053

### Cisplatin-induced upregulation of *EGR1* is suppressed in HE4-overexpressing cells

The top fifteen annotated, protein-coding genes that were differentially regulated between SKOV3-NV and SKOV3-C1 cells in the presence of cisplatin are listed in Table 2. This list excludes genes that were already differentially regulated between SKOV3-NV and SKOV3-C1 vehicle treated cells. Of these genes, early growth response 1 (*EGR1*), which is a key gene involved in regulating growth and apoptosis in a wide variety of tissues, presented as the most differentially expressed, with 4.07-fold higher expression in SKOV3-NV than SKOV3-C1 (*p* = .000201). Silencing of *EGR1* has been shown to promote resistance to cisplatin in esophageal cancer cells [14], while overexpression of this gene sensitizes ovarian cancer cells to cisplatin-induced apoptosis [15]. Thus, we speculate that suppression of *EGR1* upregulation serves as a downstream effector of HE4-mediated cisplatin resistance.

**Table 2:** Genes differentially expressed between SKOV3-NV and SKOV3-C1 (Cisplatin Treated). SKOV3-NV and SKOV3-C1 cells were treated with vehicle (DMSO) or 25 μM cisplatin for 24 h (*n* = 3/group). RNA was isolated and Affymetrix HTA 2.0 arrays were performed to determine differences in gene expression between SKOV3-NV and SKOV3-C1 cells. The top fifteen protein-coding, annotated genes with ≥1.5-fold change (*p* < 0.05) in either direction (excluding those genes that were already differentially expressed in vehicle treated cells) are listed below

Genes differentially expressed between SKOV3-NV and SKOV3-C1 (Cisplatin Treated)			
Gene symbol	Description	Fold change (NV/C1)	ANOVA *p*-value
EGR1	Early growth response 1	4.07	.000201
GDF15	Growth differentiation factor 15	2.74	.00056
ARL14	ADP-ribosylation factor-like 14	2.72	.000978
HIST2H4B	Histone cluster 2, H4b	2.63	0.005913
ZNF280A	Zinc finger protein 280A	2.16	.003044
SNAI1	Snail family zinc finger 1	2.03	.003606
DDIT3	DNA-damage-inducible transcript 3	1.98	.000087
RND1	Rho family GTPase	1.91	.003688
IDI2	Isopentenyl-diphosphate delta isomerase 2	1.91	.004807
TNF	Tumor necrosis factor	1.9	0.017979
ULBP2	UL16 binding protein 2	1.89	.001767
IL6	Interleukin 6	1.87	.000493
CCL20	Chemokine (C-C motif) ligand 20	1.86	0.000585
RAET1L	Retinoic acid early transcript 1 L	1.84	0.000244
PPP1R15A	(protein phosphatase 1, regulatory subunit 15A)	1.83	.001381
KDM6A	(lysine (K)-specific demethylase 6A)	-1.94	.006738
MYO9A	Myosin IXA	-1.94	0.002316
CCBE1	Collagen and calcium binding EGF domains 1	-1.94	0.001091
GLDC	Glycine dehydrogenase (decarboxylating)	-1.96	0.001179
RHOT1	Ras homolog family member T1	-1.96	0.001224
THBS1	Thrombospondin 1	-1.96	0.002038
APOO	Apolipoprotein O	-1.97	.001363
RNA5SP423	Ribosomal protein S6 kinase, 90 kDa, polypeptide 3	-2.05	.004154
RPS6KA3	Ribosomal protein S6 kinase, 90 kDa, polypeptide 3	-2.05	.004154
TBL1X	Transducin (beta)-like 1X-linked	-2.1	.002705
CDH6	Cadherin 6, type 2, K-cadherin (fetal kidney)	-2.11	.00019
USP9X	Ubiquitin specific peptidase 9, X-linked	-2.12	.003764
PDK3	Pyruvate dehydrogenase kinase, isozyme 3	-2.17	.001499
BRWD3	Bromodomain and WD repeat domain containing 3	-2.27	.004584
ARL14EPL	ADP-ribosylation factor-like 14 effector protein-like; laeverin	-2.33	.002723

To further analyze genome-wide differences in cisplatin response between SKOV3-NV and SKOV3-C1 cells, we also conducted the following comparisons: SKOV3-NV (vehicle vs. cisplatin) and SKOV3-C1 (vehicle vs. cisplatin). The top fifteen differentially expressed annotated, protein-coding genes in either direction are displayed in Table 3. We also included *EGR1* in this table, since it just missed the cutoff as the sixteenth gene upregulated in SKOV3-NV cells by cisplatin, but not in SKOV3-C1. Several other genes are represented in both Tables 2 and 3, including snail family zinc finger 1, (*SNAI1)*, DNA-damage-inducible transcript 3 (*DDIT3)*, ADP-ribosylation factor-like 14 (*ARL14)*, and tumor necrosis factor (*TNF*). Moreover, while there were several genes that were cisplatin-regulated in both SKOV3-NV and SKOV3-C1, the overall number of all transcripts regulated by cisplatin (+/- 1.5-fold, *p* < .05) in SKOV3-NV was much greater than in SKOV3-C1 cells (5935 vs. 1035), as represented by comparative scatter plots (Additional file 4). Even among those genes that were regulated in both SKOV3-NV and SKOV3-C1 in Table 3, there tended to be a greater degree of change in SKOV3-NV cells, especially for those genes that were upregulated by cisplatin. These observations are in agreement with the stronger cisplatin-response in SKOV3-NV cells.

**Table 3:** Genes Differentially Expressed Upon Cisplatin Treatment of SKOV3-NV and SKOV3-C1 Cells. SKOV3-NV and SKOV3-C1 cells were treated with vehicle (DMSO) or 25 μM cisplatin for 24 h (*n* = 3/group). RNA was isolated and Affymetrix HTA 2.0 arrays were performed to determine cisplatin-induced differences in gene expression in SKOV3-NV and SKOV3-C1 cells. The top fifteen annotated, protein-coding genes (*p* < 0.05) differentially expressed in either direction are listed below, as well as *EGR1*, which was the sixteenth gene change for SKOV3-NV cells

SKOV3-NV-veh vs. SKOV3-NV-cis				SKOV3-C1-veh vs. SKOV3-C1-cis			
Gene symbol	Description	Fold-change	ANOVA *p*-value	Gene symbol	Description	Fold-change	ANOVA *p*-value
PRKCA	Protein kinase C, alpha	7.21	0.000002	PRKCA	Protein kinase C, alpha	5.39	0.002139
SBF2	SET binding factor 2	6.27	4.50E-07	TMEM117	Transmembrane protein 117	5.24	0.001217
CDH6	Cadherin 6, type 2, K-cadherin (fetal kidney)	5.36	0.000009	PTPRG	Protein tyrosine phosphatase, receptor type, G	5.22	0.000633
DYM	Dymeclin	5.24	2.43E-07	PTPRM	Protein tyrosine phosphatase, receptor type, M	4.89	0.000015
DPYD	Dihydropyrimidine dehydrogenase	5.17	0.000011	MSI2	Musashi RNA-binding protein 2	4.79	0.013201
MSI2	Musashi RNA-binding protein 2	5.04	0.00001	CDKAL1	CDK5 regulatory subunit associated protein 1-like 1	4.69	0.005495
PTPRM	Protein tyrosine phosphatase, receptor type, M	4.99	0.000004	DPYD	Dihydropyrimidine dehydrogenase	4.61	0.001272
EXOC6B	Exocyst complex component 6B	4.95	0.000001	PITPNC1	Phosphatidylinositol transfer protein, cytoplasmic 1	4.56	0.000222
CDKAL1	CDK5 regulatory subunit associated protein 1-like 1	4.9	0.000013	SBF2	SET binding factor 2	4.29	0.000294
TMEM117	Transmembrane protein 117	4.81	0.000003	SAMD12	Sterile alpha motif domain containing 12	4.19	0.004401
MAML2	Mastermind-like 2 (Drosophila)	4.78	0.000041	TENM1	Teneurin transmembrane protein 1	4.13	0.000457
SPTLC3	Serine palmitoyltransferase, long chain base subunit 3	4.76	0.000016	GTDC1	Glycosyltransferase-like domain containing 1	3.99	0.007196
PTPRG	Protein tyrosine phosphatase, receptor type, G	4.67	9.73E-07	EXOC6B	Exocyst complex component 6B	3.98	0.002653
TRHDE	Thyrotropin-releasing hormone degrading enzyme	4.66	0.000011	CDH13	Cadherin 13, H-cadherin (heart)	3.83	0.000243
FTO	Fat mass and obesity associated	4.57	2.54E-07	FTO	Fat mass and obesity associated	3.69	0.009
EGR1	Early growth response 1	-3.51	0.000319	--	--	--	--
FRG2	FSHD region gene 2; FSHD region gene 2-like	-3.57	0.000156	SNAI1	(snail family zinc finger 1)	-2.16	.000605
PPP1R15A	Protein phosphatase 1, regulatory subunit 15A	-3.69	0.000047	NR4A3	Nuclear receptor subfamily 4, group A, member 3	-2.17	0.00205
SNAI1	Snail family zinc finger 1	-3.85	0.00028	PMAIP1	Phorbol-12-myristate-13-acetate-induced protein 1	-2.22	2.51E-07
HBEGF	Heparin-binding EGF-like growth factor	-4.01	0.000148	FRG2	FSHD region gene 2; FSHD region gene 2-like	-2.24	0.033969
NR4A3	Nuclear receptor subfamily 4, group A, member 3	-4.05	0.000145	GADD45A	Growth arrest and DNA-damage-inducible, alpha	-2.26	0.000042
TNFAIP3	Tumor necrosis factor, alpha-induced protein 3	-4.23	0.000031	CCL26	Chemokine (C-C motif) ligand 26	-2.37	0.023835
GADD45A	Growth arrest and DNA-damage-inducible, alpha	-4.23	0.000007	RND1	Rho family GTPase 1	-2.58	0.011645
DDIT3	DNA-damage-inducible transcript 3	-4.26	0.000017	TNFAIP3	Tumor necrosis factor, alpha-induced protein 3	-2.61	0.001029
ARL14	ADP-ribosylation factor-like 14	-4.28	0.000109	CCL20	Chemokine (C-C motif) ligand 20	-2.64	0.000022
TNF	Tumor necrosis factor	-4.38	0.000048	BIRC3	Baculoviral IAP repeat containing 3	-2.71	0.000066
CCL20	Chemokine (C-C motif) ligand 20	-4.57	0.000051	HBEGF	Heparin-binding EGF-like growth factor	-2.96	0.000133
RND1	Rho family GTPase 1	-4.61	0.000069	ATF3	Activating transcription factor 3	-2.96	0.000575
ATF3	Activating transcription factor 3	-6.24	0.000007	IL1A	Interleukin 1, alpha	-3.36	0.00006
ZNF280A	Zinc finger protein 280A	-6.28	0.000082	ZNF280A	Zinc finger protein 280A	-3.78	0.000008
IL8	Interleukin 8	-10.71	0.000041	IL8	Interleukin 8	-6.41	0.000002

**Fig. 4:**
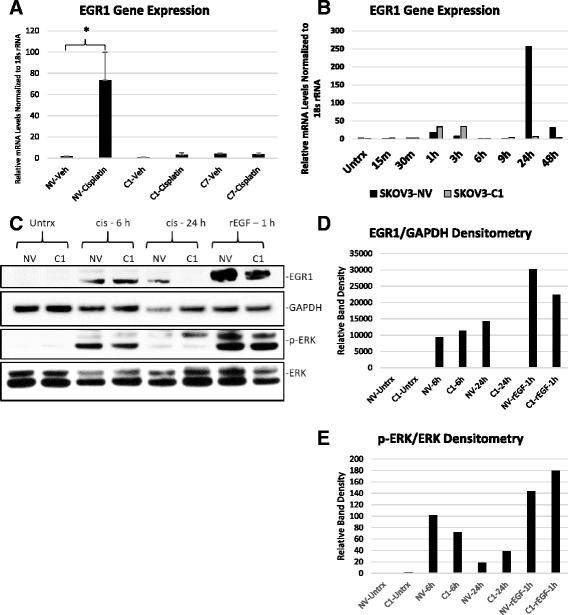
EGR1 mRNA and protein upregulation is suppressed in HE4-overexpressing clones. **a** Quantitative RT-PCR validation of microarray using RNA from SKOV3-NV, SKOV3-C1, and SKOV3-C7 cells treated with vehicle (DMSO) or 25 μM cisplatin. Error bars represent standard deviation of three biological replicates, *p < .05, **p < .005. **b** Timecourse analysis of *EGR1* expression following treatment of SKOV3-NV and SKOV3-C1 cells with 25 μM cisplatin. **c** EGR1, phosphorylated ERK, and total ERK protein levels following treatment with vehicle (DMSO), 25 μM cisplatin, or 10 ng/mL rEGF (positive control) were determined by western blot. GAPDH was used as a loading control. **d**-**e** Densitometry analysis of EGR1 (normalized to GAPDH) and phospho-ERK/ERK ratio, from western blot in (**c**)

Because *EGR1* was most the differentially expressed gene between SKOV3-NV and SKOV3-C1 treated with cisplatin, and has known roles in cisplatin response, we validated its differential regulation by qRT-PCR using SKOV3-NV, SKOV3-C1, and SKOV3-C7 RNA exposed to vehicle (DMSO) or 25 μM cisplatin for 24 h (Fig. 4a). Next, because *EGR1* is an early response gene reported to be upregulated as early as 15–30 min following exposure to a stimulant [16, 17], we performed a time course analysis of *EGR1* expression following cisplatin treatment of SKOV3-NV and SKOV3-C1 cells. Interestingly, an early upregulation of *EGR1* occurred in both SKOV3-NV and SKOV3-C1 cells beginning at 15 min after cisplatin treatment, which peaked between 1 and 3 h post-treatment and diminished by 6 h. However, the second more robust increase in *EGR1* expression observed at 24 h in SKOV3-NV cells was dramatically suppressed in SKOV3-C1 cells (Fig. 4b). A similar trend was noted in EGR1 protein levels. While EGR1 protein was increased at 6 h in both SKOV3-NV and SKOV3-C1 cells treated with 25 μM cisplatin, only SKOV3-NV cells maintained higher levels of EGR1 by 24 h (Fig. 4c-d). To confirm the intrinsic ability of SKOV3-C1 cells to upregulate *EGR1*, both SKOV3-NV and SKOV-C1 cells were treated with 10 ng/ml human recombinant epidermal growth factor (rEGF) to activate the EGFR signaling pathway, a known positive regulator of *EGR1* gene expression [18, 19]. We observed a robust increase in *EGR1* at 1 h after treatment in both SKOV3-NV and SKOV3-C1 cells (Fig. 4c-d), which is in agreement with a previous study using the same dose and time point [18]. These data indicate that HE4-overexpressing cells are capable of upregulating *EGR1*, but some mechanism is activated to suppress its later upregulation in response to cisplatin. Activation of ERK in response to cisplatin and rEGF was comparable between SKOV3-NV and SKOV3-C1 cells (Fig. 4c and e).

### HE4 promotes deregulation of mitogen activated protein kinase (MAPK) signaling

**Fig. 5:**
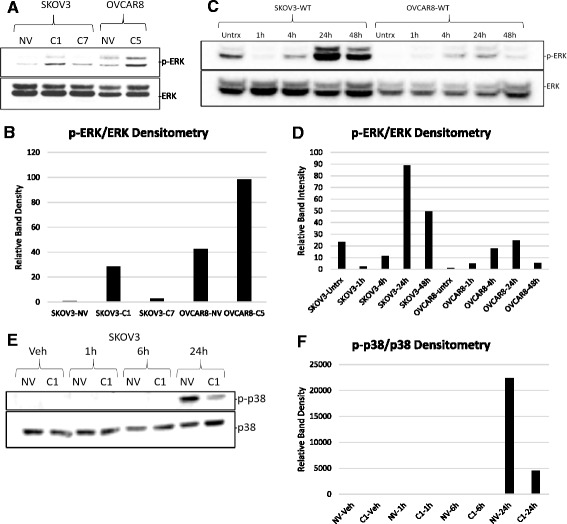
HE4 promotes deregulation of mitogen activated protein kinase (MAPK) signaling. **a** Protein was collected from SKOV3-NV, SKOV3-C1, and SKOV3-C7 cells and OVCAR8-NV and OVCAR8-C5 cells and western blot performed for phosphorylated ERK and total ERK. **b** Densitometry analysis of phospho-ERK/ERK ratio, from western blot in (**a**). **c** SKOV3-WT and OVCAR8-WT cells were treated with 20 nM recombinant HE4 for the indicated times, or left untreated. Protein was collected and western blot performed to determine levels of phosphorylated ERK and total ERK. **d** Densitometry analysis of phospho-ERK/ERK ratio, from western blot in (**c**). **e** SKOV3-NV and SKOV3-C1 cells were treated with vehicle (DMSO) or 25 μM cisplatin for the indicated times and western blot performed to detect levels of phospho-p38 and total p38. **f** Densitometry analysis of phospho-p38/p38 ratio, from western blot in (**e**)

Because MAPKs transcriptionally activate *EGR1* expression by phosphorylating the *EGR1* transcription factor ELK-1 [19–21], we assessed levels of phosphorylated extracellular signal-regulated kinase (ERK) in SKOV3-NV, SKOV3-C1, SKOV3-C7, OVCAR8-NV, and OVCAR8-C5 cell lines, and observed that levels of phospho-ERK were higher in SKOV3 and OVCAR8 HE4-overexpressing clones than in SKOV3-NV cells (Fig. 5a-b). In addition, when SKOV3 wild type (WT) and OVCAR8-WT cells were treated with 20 nM human recombinant HE4 (rHE4) protein, levels of phospho-ERK were dramatically affected (Fig. 5c-d). In SKOV3-WT cells, phospho-ERK was almost completely obliterated as early as 1 h after treatment, but levels were restored to above baseline by 24 h. Conversely, phospho-ERK continuously increased from 1 h to 24 h in OVCAR8-WT cells. These differential results between SKOV3 and OVCAR8 cells suggest time-dependent and cell type-specific effects of HE4 on growth factor signaling, but highlight the fact that HE4 has a rapid and dramatic effect on kinase activation, which could in turn affect regulation of *EGR1* in response to cisplatin.

Although we see a significant effect of rHE4 on phospho-ERK levels, we were unable to detect differences in ERK activation between SKOV3-NV and SKOV3-C1 in response to 6 h cisplatin treatment, as previously shown. Moreover, by 24 h (the timepoint where we see the greatest rise in *EGR1*), phospho-ERK levels are diminished in both SKOV3-NV and SKOV3-C1 cells (Fig. 4c and e). Since p38—another MAPK known to regulate EGR1 transcription [20]—is also a key player in promoting apoptosis in response to cisplatin, we examined levels of phospho-p38 in SKOV3-NV and SKOV3-C1 clones following treatment with 25 μM cisplatin for 1, 6, and 24 h. Interestingly, p38 was robustly activated at 24 h in SKOV3-NV cells following cisplatin treatment, but this response was blunted in SKOV3-C1 cells (Fig. 5e-f). We also observed similar results comparing p38 activation in SKOV3-NV, SKOV3-C1, and SKOV3-C7 cells using a higher dose of cisplatin (80 μM), with robust activation noted at 6 h only in SKOV3-NV cells (Additional file 5A and B). We did observe a small suppression of ERK activation beginning at 1 h in HE4-overexpressing SKOV3 clones treated with this high dose of cisplatin, which was most apparent at 6 h after treatment, suggesting there may be subtle differences in how ERK is activated in HE4-overexpressing clones that only become apparent with high dose treatment (Additional file 5A and C).

### HE4 modulates tubulin levels and MAPT gene expression

**Fig. 6:**
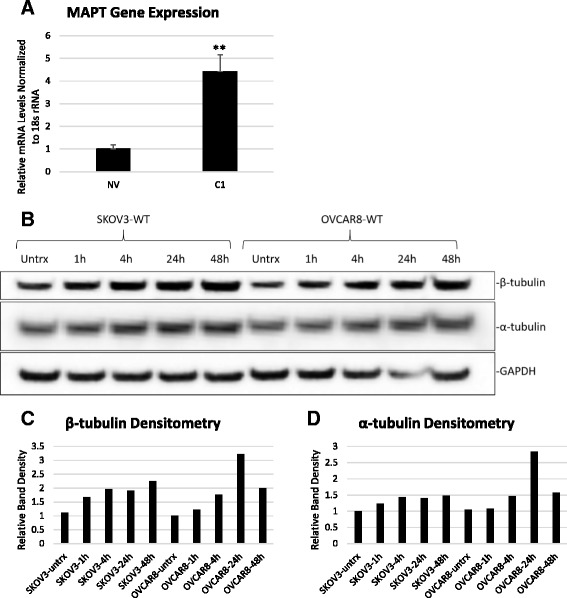
HE4 promotes tubulin deregulation in SKOV3 and OVCAR8 cells. **a** Quantitative RT-PCR of *MAPT* in SKOV3-NV and SKOV3-C1 cells, normalized to 18 s rRNA. **p < .05 **b** SKOV3-WT and OVCAR8-WT cells were left untreated or treated with 20 nM recombinant HE4, and protein was collected at the indicated timepoints. Levels of β-tubulin and α-tubulin were determined by western blot, with GAPDH used as a loading control. **c**-**d** Densitometry analysis of β-tubulin and α-tubulin, normalized to GAPDH, from western blot in (**b**)

One of the genes we found to be differentially expressed between SKOV3-NV and SKOV3-C1 (vehicle treated) in our microarray analysis was microtubule associated protein tau (*MAPT*). *MAPT*, which is associated with paclitaxel resistance in several cancers including EOC [22–28], was more highly expressed in SKOV3-C1 cells than in SKOV3-NV cells (2.47-fold, *p* = .00702) (Additional file 2). We validated these results by qPCR, finding 4.42-fold higher levels in SKOV3-C1 than SKOV3-NV (*p* = .00446) (Fig. 6a). Since higher levels of *MAPT* together with β-tubulin correlate with paclitaxel resistance in gastric cancer [23], we sought to determine if HE4 affects β-tubulin levels and microtubule stability. Treatment of SKOV3-WT and OVCAR8-WT cells with 20 nM rHE4 produced a continuous increase in β-tubulin levels between 1 h and 48 h after treatment (Fig. 6b and c). Levels of α-tubulin also increased over time, similar to what was seen with β-tubulin (Fig. 6b and d). Collectively, these data suggest that HE4 may in part promote paclitaxel resistance by affecting microtubule levels or stability.

## Discussion

The ways in which HE4 affects chemoresistance are likely multi-factorial and cell type-specific. In cells that already display a high degree of chemoresistance due to other mechanisms, such as OVCAR8 cells, the effect of increasing levels of HE4 may be minimal. In contrast, in cells with low levels of HE4 that are not as chemoresistant, increasing the level of HE4 may produce dramatic changes in apoptotic response to drug treatment, as we have observed in SKOV3 cells.

Human HE4 purified from seminal fluid has been described to possess cross-class protease activity [4]; however, that study reports seminal fluid HE4 to exist as a trimer migrating at 42 kDa under non-reducing conditions and 14 kDa under SDS-PAGE reducing conditions. It is possible that HE4 from different tissues may preferentially exist in different forms, thus possessing different functions. Indeed, in our lab, we observe HE4 migrating on SDS-PAGE under reducing conditions as a 25 kDa protein, which is in agreement with a study by Drapkin et al. showing glycosylated HE4 migrating at 25 kDa in CaOV3 and OVCAR5 cells [29]. This diversity in size may result in different enzymatic activities of HE4. If HE4 does modulate protease activity, it logically will affect a wide variety of cellular functions, since proteases are essential for many biological processes including growth factor signaling. For example, hepatocyte growth factor (HGF) [30], transforming growth factor beta (TGFβ) [31], and certain members of the platelet-derived growth factor (PDGF) family [32] are activated by proteases. Our data would suggest that anti-proteases may also serve a function in activating growth factor cascades in some cases.

In our current study, we have observed a variety of differences between SKOV3-NV and HE4-overexpressing cells in how they respond to cisplatin and paclitaxel. EGR1 is a transcription factor that is induced by a variety of stimuli or stresses, including growth factors, hormones, ionizing radiation, and chemotherapeutic drugs [16, 19, 33–37]. It has been shown to regulate differentiation, proliferation, and apoptosis in cell type-specific manners by promoting expression of several genes, including *TP53* (p53), *BCL2*, *PTEN*, *IGF2*, *PDGF*, *VEGF*, *TGFB1*, and *TNF* [18]. In our study, at 24 h after cisplatin treatment, *EGR1* is upregulated in SKOV3-NV cells, but not in SKOV3-C1 or SKOV3-C7 cells. EGR1 is a transcriptional regulator of growth differentiation factor 15 (*GDF15*) [38], which was also upregulated in SKOV3-NV by cisplatin but not in SKOV3-C1 cells. While *GDF15* has been linked to platinum resistance in pancreatic cancer [39] and ovarian cancer [40], it has also been shown to be a common platinum-responsive gene [41, 42], and was identified as a potential serum marker for cisplatin-response of ovarian cancer cells [43]. Thus, the fact that it is not upregulated by cisplatin in SKOV3-C1 cells is also indicative of their dampened cisplatin resistance. Another gene, DNA-damage-inducible transcript 3 (*DDIT3*), was also cisplatin-induced in SKOV3-NV cells, but not SKOV3-C1 cells. Suppression of *DDIT3* mRNA upregulation has been shown to be involved in chemoresistance of malignant pleural mesothelioma cells [44], further suggesting that multiple mechanisms may play a role in HE4-mediated chemoresistance. Lastly, tumor necrosis factor (TNF), whose gene is also EGR1-regulated [18], activates MAPKs and induces apoptosis [45]; thus, its lack of cisplatin-mediated upregulation in SKOV3-C1 cells could also contribute to suppression of pro-apoptotic signaling and cisplatin response in these cells.

*EGR1* gene expression is known to be regulated by activated MAPKs, including phospho-ERK [19, 21] and phospho-p38 [20]. Although we observe comparable increases in phospho-ERK levels in SKOV3-NV and SKOV3-C1 cells treated with 25 μM cisplatin, it is possible that HE4 affects the activity of phospho-ERK, leading to suppression of EGR1. Indeed, our results showing subtle suppression of ERK activation in HE4-overexpressing clones treated with high dose cisplatin (80 μM), as well as an effect of rHE4 on ERK activation in SKOV3-WT and OVCAR8-WT cells, support this hypothesis. HE4-mediated suppression of p38 appears to be more straightforward. We did not observe any effect of rHE4 on phospho-p38 levels (data not shown), but we did see more robust activation of p38 in SKOV3-NV cells than in SKOV3-C1 or SKOV3-C7 cells in response to two different doses of cisplatin (25 μM and 80 μM), confirming that some elements of MAPK signaling are deregulated in HE4-overexpressing cells. This suppression of p38 activation could play a role in the suppression of cisplatin-induced upregulation of *EGR1.*

Another significant effect that we report here is the increase in α-tubulin and β-tubulin levels in SKOV3-WT and OVCAR8-WT cells treated with rHE4. Interestingly, tubulins have been reported to be the target of the serine protease HtrA1 [46]; therefore, if HE4 inhibits serine protease activity, tubulins may accumulate. We postulate that the increase in tubulin levels we observe is not due to a transcriptional effect, since we did not detect an increase in any tubulin gene in OVCAR8-WT cells treated with 20 nM rHE4 for 6 h (data not shown). However, as we show here, we do see an increase in gene expression levels of *MAPT* in SKOV3-C1 cells compared to SKOV3-NV, which together with a putative stabilization of tubulin protein could influence paclitaxel resistance. Several lines of evidence connect β-tubulin and *MAPT* to taxane resistance. In addition to the correlation between *MAPT* and β-tubulin III levels and paclitaxel resistance in gastric cancer [23], downregulation of *MAPT* was also shown to improve taxane response in breast cancer cell lines [24] and in ovarian cancer three-dimensional collagen I matrix culture [22]. Clinically, expression of the tau protein is associated with worse survival of taxane-treated breast cancer patients [28]. Since tau proteins are responsible for stabilizing microtubules [47], higher levels of MAPT could affect the polymerization of microtubules by paclitaxel.

In addition to differences in how HE4-overexpressing cells respond to cisplatin, intrinsic differences between SKOV3-NV and SKOV3-C1 cells could also contribute to resistance, or could indicate other biological functions of HE4. For example, *AKT3*, which is more highly expressed in SKOV3-C1 cells, has been shown to promote cisplatin resistance in SKOV3 and OVCAR3 cells [48]. Another gene of interest that is upregulated in SKOV3-C1 cells is *SEPT3*. The septin family of GTP-binding proteins polymerize to form cytoskeletal filaments [49], further implicating HE4’s putative involvement in cytoskeleton organization. Several genes that appear upregulated in HE4-overexpressing cells are in agreement with the pro-proliferative role described for HE4 [7, 11, 50], including *AKT3*, Ras-related GTP binding D (*RRAGD*), and glypican 4 (*GPC4*).

Several other future directions remain to clarify how HE4 promotes collateral resistance to platinum and taxane therapies. Further clarification of precisely how HE4 affects cell signaling, including MAPK signaling, in diverse cell lines is necessary. Confirmation of HE4’s anti-protease function in ovarian cancer cells would be useful in this regard. Moreover, additional information is needed on how HE4 affects tubulin organization and dynamics. Lastly, although we do not have any preliminary data to suggest that there are differences in drug uptake or clearance between SKOV3-NV and SKOV3-C1 cells, it would be necessary to measure intracellular drug levels at various time points post-treatment before ruling this out as another contributing mechanism to chemoresistance. Future studies should also address whether HE4 promotes resistance to other commonly used ovarian cancer treatments such as doxorubicin.

## Conclusions

In summary, we have demonstrated that HE4 promotes collateral resistance to cisplatin and paclitaxel in SKOV3 and OVCAR8 ovarian cancer cells. Importantly, downregulating HE4 gene transcription using Double Nickase CRISPR/Cas technology results in reduced secretion of HE4 in SKOV3-C1 cells, and partially reverses chemoresistance. In this study, we show that there are likely multiple factors that contribute to HE4-mediated chemoresistance, although two emerging factors that we present here for the first time are deregulation of MAPK signaling and altered tubulin dynamics. As we gain a better understanding of how HE4 contributes to resistance to various chemotherapies, monitoring HE4 levels before, during, and after chemotherapy cycles may help predict responses and dictate treatment decisions. Furthermore, small molecules or antibodies targeting HE4 may enhance the efficacy of first- or second-line therapeutics and reduce the development of resistance.

## Availability of datasets

The microarray dataset supporting the conclusions of this article is included within its additional file.

## Additional files

Additional file 1: HE4 overexpression enhances resistance to cisplatin and paclitaxel in OVCAR8 cells. OVCAR8-null vector (NV) and OVCAR8-HE4 clone 5 (C5) cells were treated with 0-500 μM cisplatin for 24 h (A) or 0-5 nM paclitaxel for 48 h (B), at which time the cells were subjected to MTS assay to measure viability. Error bars represent standard deviation of technical replicates. (C) OVCAR8-NV and OVCAR8-C5 cells were treated with vehicle (DMSO) or 100 μM cisplatin, and protein was collected at 24 h after treatment. Western blot was performed to detect levels of PARP and cleaved PARP. (D) Densitometry analysis of PARP and cleaved PARP normalized to β-tubulin levels, from western blot in (C). (PPTX 90 kb) Additional file 2: All genes differentially regulated between SKOV3-NV and SKOV3-C1 (vehicle and cisplatin treated) 1.5-fold in either direction (*p* < 0.05). (XLSX 209 kb) Additional file 3: Top 10 DAVID annotation clusters of genes differentially expressed between SKOV3-NV and SKOV3-C1. Analysis of all genes with fold-change ≥1.5 (*p* < .05) in either direction. (XLSX 10 kb) Additional file 4: Reduced transcriptional regulation by cisplatin in SKOV3-C1 cells. Scatterplots of all transcripts upregulated (green) or downregulated (red) by cisplatin in SKOV3-NV (A) and SKOV3-C1 (B) were included. Gray area represents filtered genes (fold-change <1.5 in either direction, *p* > .05). (PPTX 215 kb) Additional file 5: Overexpression of HE4 suppresses cisplatin-mediated activation of p38 and ERK. (A) SKOV3-NV, SKOV3-C1, and SKOV3-C7 cells were treated with vehicle (DMSO) or 80 μM cisplatin for the indicated times. Western blot was performed to detect levels of phospho-p38 and total p38. (B) Densitometry analysis of p-p38/p38 ratio from (A). (C) Densitometry analysis of phospho-ERK/ERK ratio from (A). (PPTX 443 kb)

## Abbreviations

C1, C5, C7: clone 1, clone 5, clone 7
CRISPR: clusters of regularly interspaced short palindromic repeats
EOC: epithelial ovarian cancer
EGFR: epidermal growth factor receptor
ERK: extracellular signal-regulated kinase
HGSC: high grade serous ovarian cancer
MAPK: mitogen activated protein kinase
NV: null vector
PARP: poly ADP ribose polymerase
PLD: pegylated liposomal doxorubicin
qRT-PCR: quantitative reverse transcription polymerase chain reaction
rEGF: recombinant human epidermal growth factor
rHE4: recombinant human epididymis protein 4
RMI: risk of malignancy index
ROMA: risk of ovarian malignancy algorithm
WAP: whey acidic protein
WT: wild type
