# Supplementary material for: HE4 promotes collateral resistance to cisplatin and paclitaxel in ovarian cancer cells
Source: J Ovarian Res. 2016 May 17;9:28. doi: 10.1186/s13048-016-0240-0 (PMC4869286; doi:10.1186/s13048-016-0240-0)
Supplement: Additional file 1: — HE4 overexpression enhances resistance to cisplatin and paclitaxel in OVCAR8 cells. OVCAR8-null vector (NV) and OVCAR8-HE4 clone 5 (C5) cells were treated with 0-500 μM cisplatin for 24 h (A) or 0-5 nM paclitaxel for 48 h (B), at which time the cells were subjected to MTS assay to measure viability. Error bars represent standard deviation of technical replicates. (C) OVCAR8-NV and OVCAR8-C5 cells were treated with vehicle (DMSO) or 100 μM cisplatin, and protein was collected at 24 h after treatment. Western blot was performed to detect levels of PARP and cleaved PARP. (D) Densitometry analysis of PARP and cleaved PARP normalized to β-tubulin levels, from western blot in (C). (PPTX 90 kb) [file 13048_2016_240_MOESM1_ESM.pptx]

## Slide 1
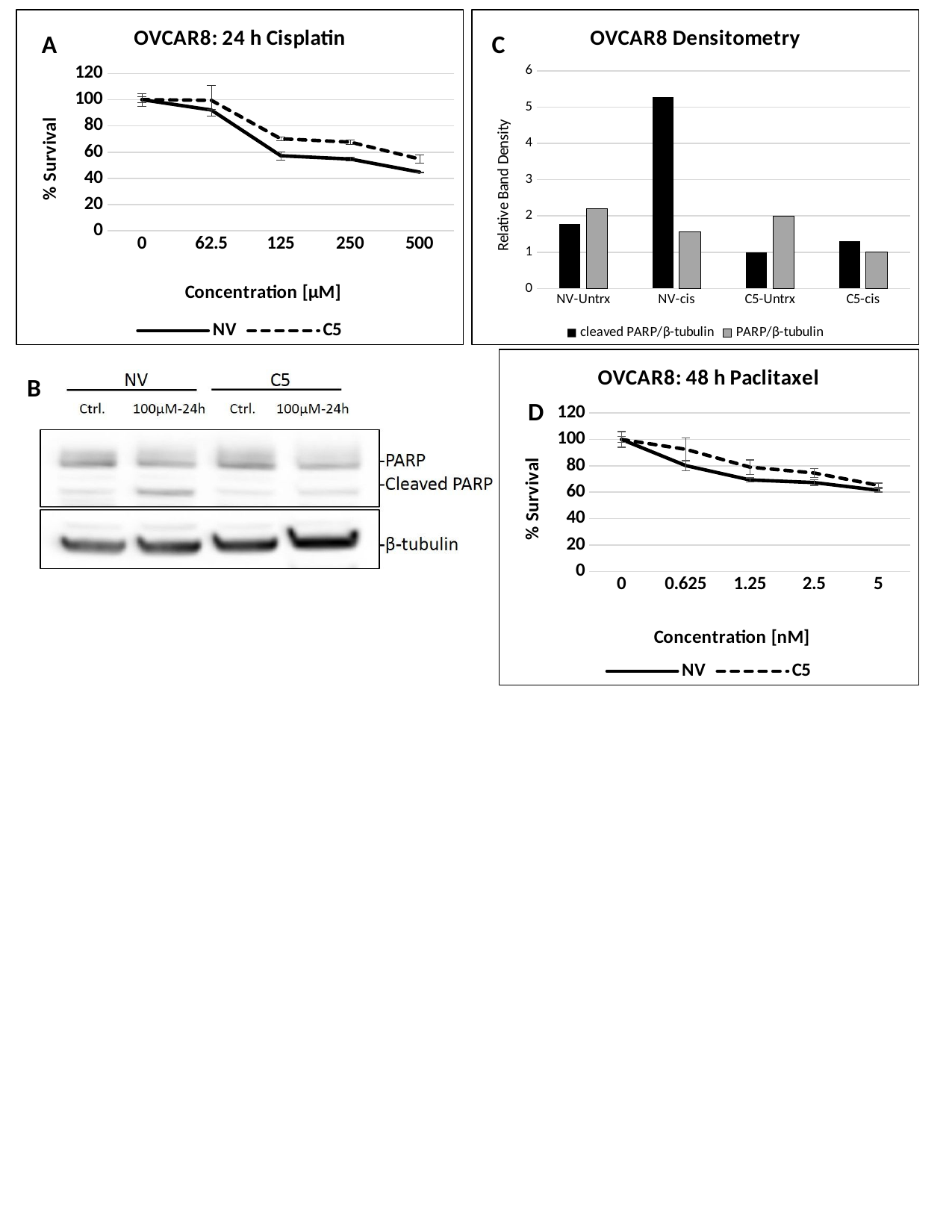

### Chart: OVCAR8: 24 h Cisplatin
| Category | NV | C5 |
|---|---|---|
| 0 | 100.0 | 100.0 |
| 62.5 | 92.08523592085236 | 99.47478991596641 |
| 125 | 57.22983257229832 | 70.16806722689076 |
| 250 | 54.718417047184175 | 67.64705882352942 |
| 500 | 44.748858447488594 | 54.726890756302524 |
### Chart: OVCAR8 Densitometry
| Category | cleaved PARP/β-tubulin | PARP/β-tubulin |
|---|---|---|
| NV-Untrx | 1.7821965131004913 | 2.198889126138857 |
| NV-cis | 5.291810984989175 | 1.5634960914031795 |
| C5-Untrx | 1.0 | 1.9867961051531753 |
| C5-cis | 1.312704333347948 | 1.0 |A
C
### Chart: OVCAR8: 48 h Paclitaxel
| Category | NV | C5 |
|---|---|---|
| 0 | 100.0 | 100.0 |
| 0.625 | 80.16893132574366 | 92.49190938511327 |
| 1.25 | 69.2985677561513 | 79.02912621359224 |
| 2.5 | 67.31546088872567 | 74.56310679611651 |
| 5 | 61.47631289019463 | 65.24271844660194 |
B
D
