# Supplementary material for: HE4 promotes collateral resistance to cisplatin and paclitaxel in ovarian cancer cells
Source: J Ovarian Res. 2016 May 17;9:28. doi: 10.1186/s13048-016-0240-0 (PMC4869286; doi:10.1186/s13048-016-0240-0)
Supplement: Additional file 4: — Reduced transcriptional regulation by cisplatin in SKOV3-C1 cells. Scatterplots of all transcripts upregulated (green) or downregulated (red) by cisplatin in SKOV3-NV (A) and SKOV3-C1 (B) were included. Gray area represents filtered genes (fold-change <1.5 in either direction, p > .05). (PPTX 215 kb) [file 13048_2016_240_MOESM4_ESM.pptx]

## Slide 1
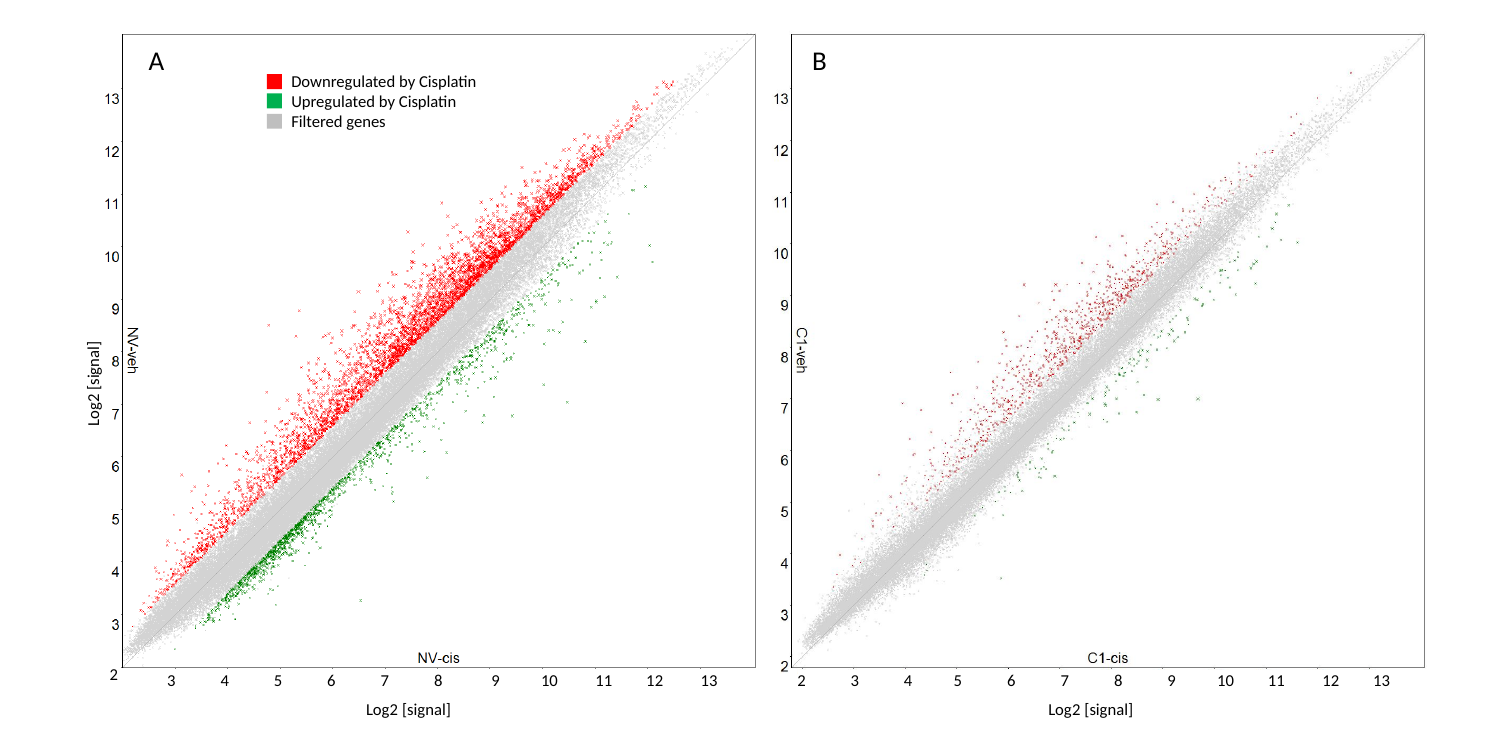

A
B
Downregulated by Cisplatin
Upregulated by Cisplatin
Filtered genes
Log2 [signal]
2
2 3 4 5 6 7 8 9 10 11 12 13
3 4 5 6 7 8 9 10 11 12 13
Log2 [signal]
Log2 [signal]
