# Supplementary material for: HE4 promotes collateral resistance to cisplatin and paclitaxel in ovarian cancer cells
Source: J Ovarian Res. 2016 May 17;9:28. doi: 10.1186/s13048-016-0240-0 (PMC4869286; doi:10.1186/s13048-016-0240-0)
Supplement: Additional file 5: — Overexpression of HE4 suppresses cisplatin-mediated activation of p38 and ERK. (A) SKOV3-NV, SKOV3-C1, and SKOV3-C7 cells were treated with vehicle (DMSO) or 80 μM cisplatin for the indicated times. Western blot was performed to detect levels of phospho-p38 and total p38. (B) Densitometry analysis of p-p38/p38 ratio from (A). (C) Densitometry analysis of phospho-ERK/ERK ratio from (A). (PPTX 443 kb) [file 13048_2016_240_MOESM5_ESM.pptx]

## Slide 1
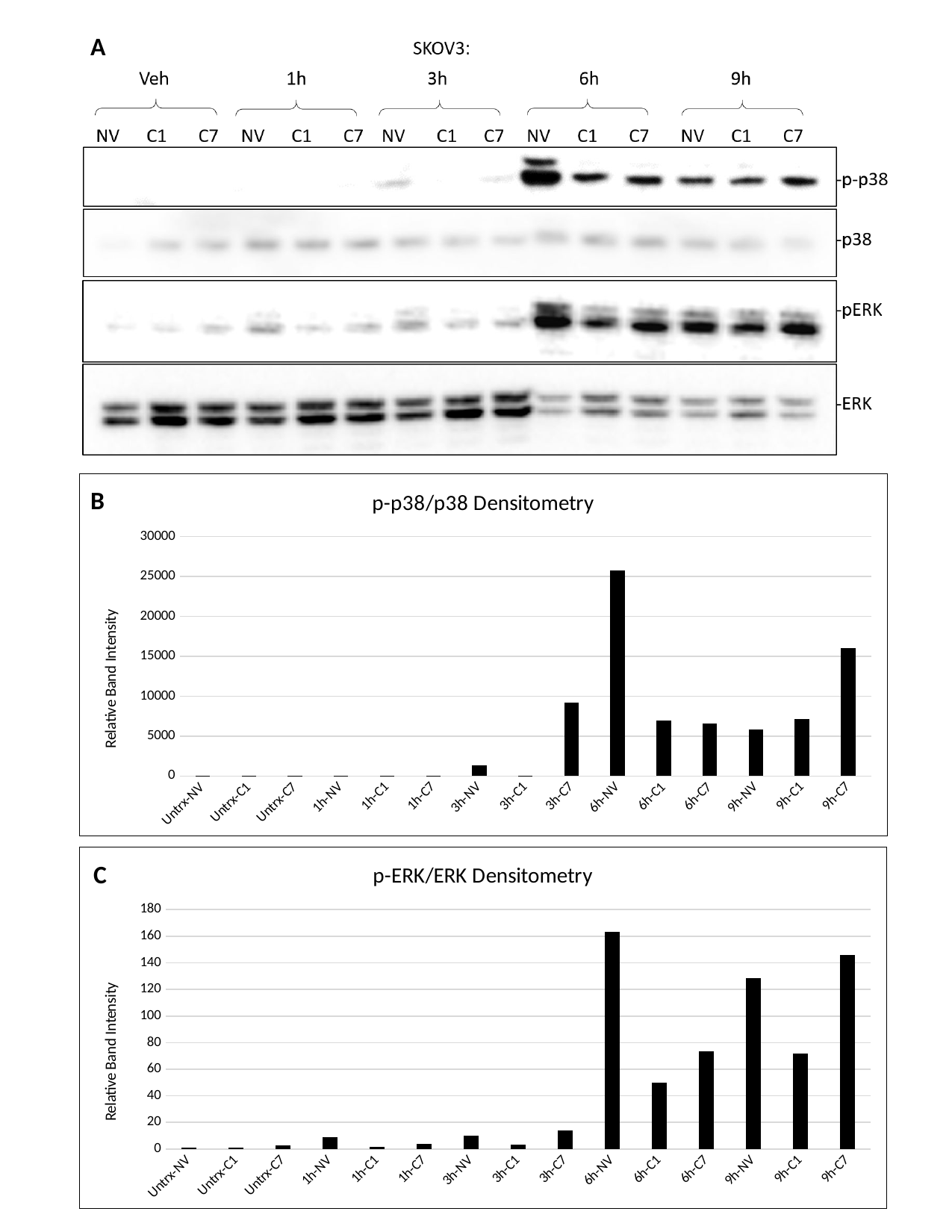

A
### Chart: p-p38/p38 Densitometry
| Category | p-p38/p38 |
|---|---|
| Untrx-NV | 10.661238516502213 |
| Untrx-C1 | 2.5110094242851653 |
| Untrx-C7 | 1.7265358615658264 |
| 1h-NV | 1.0 |
| 1h-C1 | 1.2826556827838937 |
| 1h-C7 | 1.2737022077833389 |
| 3h-NV | 1298.8709178344022 |
| 3h-C1 | 1.8723284999444278 |
| 3h-C7 | 9235.648214815368 |
| 6h-NV | 25741.077309764198 |
| 6h-C1 | 6944.0513300959765 |
| 6h-C7 | 6605.62312365691 |
| 9h-NV | 5862.591890627073 |
| 9h-C1 | 7098.098364885119 |
| 9h-C7 | 16047.874704200127 |B
### Chart: p-ERK/ERK Densitometry
| Category | p-ERK/ERK |
|---|---|
| Untrx-NV | 1.0 |
| Untrx-C1 | 1.0219563334670656 |
| Untrx-C7 | 2.6989729638999633 |
| 1h-NV | 8.762938530656818 |
| 1h-C1 | 1.7755568166784605 |
| 1h-C7 | 3.6334420035028683 |
| 3h-NV | 10.147512689209881 |
| 3h-C1 | 3.0894186232615954 |
| 3h-C7 | 13.774067424463665 |
| 6h-NV | 163.43220434772113 |
| 6h-C1 | 49.73803728104416 |
| 6h-C7 | 73.54216135389204 |
| 9h-NV | 128.2310725050884 |
| 9h-C1 | 71.51458655414507 |
| 9h-C7 | 146.18259880634966 |C
